# Immune Cell and Adipose Gene Expression Reprogramming in Juvenile Monkeys Born to Health and Weight-Diverse Mothers

**DOI:** 10.1093/geroni/igab046.2545

**Published:** 2021-12-17

**Authors:** Alistaire Ruggiero, Masha Block, Ravichandra Vemuri, Darla DeStephanis, Swapan Das, Kylie Kavanagh

**Affiliations:** Wake Forest School of Medicine, Winston-Salem, North Carolina, United States

## Abstract

Over 93 million Americans are obese and 66 million suffer from metabolic disease. Roughly 40% of obese people do not have metabolic abnormalities (metabolically health obese [MHO]), while approximately 15% of lean do (metabolically unhealthy lean [MUL]). African green monkeys (AGMs) demonstrate naturally occurring obesity and metabolic syndrome (MetS) without diet manipulation, and MetS criteria are heritable. Age-matched maternal AGMs were classified by adjusted MetS criteria ([n=44]; waist >40cm, fasting glucose (FG) >100 mg/dL, SBP/DBP >135/85mmHg, and HDL-c <50mg/dL) and classified as metabolically healthy lean (MHL), MHO, MUL or metabolically unhealthy obese (MUO). Age, weight and sex-matched pre-pubertal juvenile offspring from these mothers were additionally selected (n=9-11/group; ages=1.1-3.4years) for evaluation. We assessed monocyte subtypes by flow cytometry, and subcutaneous adipose gene expression patterns by RNAseq. Non-classical monocytes were increased in obese and unhealthy mothers (MHO p=0.02, MUL p=0.003, MUO p=0.00002) compared to MHL. MUL and MUO juvenile offspring also had more non-classical monocytes compared to MHL (p=0.05 and p=0.07). Monocyte chemoattractant protein-1 (MCP)-1 was measured in plasma and found to be elevated in MUO juveniles (p=0.02). Patterns of increased cytokine and extracellular matrix gene expression were seen in MUL and MUO juveniles’ adipose (6-7/group), mirroring obese and unhealthy mothers’ adipose gene expression patterns. Maternal health and obesity influence offspring immune cells and adipose gene expression prior to weight gain and metabolic disease onset. Our data underscore maternal monocyte and adipose profiles as inherited phenotypes that present prior to adipose expansion and may be targets to improve intergenerational health trajectories.

